# Impact of vector control interventions on malaria transmission intensity, outdoor vector biting rates and *Anopheles* mosquito species composition in Tororo, Uganda

**DOI:** 10.1186/s12936-019-3076-4

**Published:** 2019-12-27

**Authors:** Alex K. Musiime, David L. Smith, Maxwell Kilama, John Rek, Emmanuel Arinaitwe, Joaniter I. Nankabirwa, Moses R. Kamya, Melissa D. Conrad, Grant Dorsey, Anne M. Akol, Sarah G. Staedke, Steve W. Lindsay, James P. Egonyu

**Affiliations:** 1 Infectious Diseases Research Collaboration, Kampala, Uganda; 2 Department of Zoology, Entomology and Fisheries Sciences, College of Natural Sciences, Makerere University, Kampala, Uganda; 3 Institute for Health Metrics & Evaluation, University of Washington, Seattle, WA USA; 4 Department of Medicine, Makerere University College of Health Sciences, Kampala, Uganda; 5 Department of Medicine, University of California San Francisco, San Francisco, CA USA; 6 Faculty of Infectious and Tropical Diseases, London School of Hygiene and Tropical Medicine, London, UK; 7 Department of Biosciences, Durham University, Durham, UK; 8 International Center of Insect Physiology and Ecology, Nairobi, Kenya

**Keywords:** Indoor residual spraying, Long-lasting insecticide nets, Malaria vector, Transmission, Biting rates

## Abstract

**Background:**

Long-lasting insecticidal nets (LLINs) and indoor residual spraying of insecticide (IRS) are widely recommended for the prevention of malaria in endemic regions. Data from human landing catches provide information on the impact of vector control on vector populations. Here, malaria transmission indoors and outdoors, before and after mass deployment of LLINs and IRS in Uganda was compared.

**Methods:**

The study took place in Tororo district, a historically high transmission area where universal LLIN distribution was conducted in November 2013 and May 2017 and 6 rounds of IRS implemented from December 2014 to July 2018. Human landing catches were performed in 8 houses monthly from October 2011 to September 2012 (pre-intervention period) and every 4 weeks from November 2017 to October 2018 (post-intervention period). Mosquitoes were collected outdoors from 18:00 to 22:00 h and indoors from 18:00 to 06:00 h. Female *Anopheles* were tested for the presence of *Plasmodium falciparum* sporozoites and species identification performed using gross dissection and polymerase chain reaction (PCR).

**Results:**

The interventions were associated with a decline in human biting rate from 19.6 to 2.3 female *Anopheles* mosquitoes per house per night (p < 0.001) and annual entomological inoculation rate from 129 to 0 infective bites per person per year (p < 0.001). The proportion of mosquitoes collected outdoors increased from 11.6 to 49.4% (p < 0.001). Prior to the interventions the predominant species was *Anopheles gambiae* sensu stricto (s.s.), which comprised an estimated 76.7% of mosquitoes. Following the interventions, the predominant species was *Anopheles arabiensis,* which comprised 99.5% of mosquitoes, with almost complete elimination of *An. gambiae* s.s. (0.5%).

**Conclusions:**

Mass distribution of LLINs and 6 rounds of IRS dramatically decreased vector density and sporozoite rate resulting in a marked reduction in malaria transmission intensity in a historically high transmission site in Uganda. These changes were accompanied by a shift in vector species from *An. gambiae* s.s. to *An. arabiensis* and a relative increase in outdoor biting.

## Background

Remarkable progress in malaria control over the last decade has been attributed to massive deployment of malaria control interventions including long-lasting insecticidal nets (LLINs), indoor residual spraying (IRS), and case management with artemisinin-based combination therapy (ACT) [1–3]. Studies of multiple malaria indicators in Tororo, Uganda, documented dramatic reductions in the incidence of malaria, prevalence of parasitaemia, test positivity rate (TPR), and annual entomological inoculation rates (aEIR) that began after IRS spraying commenced in December 2014 [4–6]. Prior to that, only a change in TPR had been observed in the months after a mass LLIN distribution in November 2013 [6]. The entomological data from Tororo suggests the effectiveness of vector-control interventions may have been modified by insecticide resistance [7]. Here, changes in vector populations and behaviour were investigated using human landing catches before and after initiation of vector control.

Responses to vector control can be affected by coverage of the intervention, by the composition of vector species and by insecticide resistance [8, 9]. Changes in the species composition of vector populations are commonly observed in response to vector-control interventions [10–12]. The changes in composition commonly occur when the vector species that are more sensitive to a specific vector-control measure become less common, leaving vector species that are less sensitive. These changes in composition are most obvious when the dominant species is reduced, although minor vectors may also increase, decrease or remain unchanged.

Changes in mosquito physiology and behaviour result to ineffectiveness vector-control interventions [13–15]. Physiological resistance involves changes in the sensitivity to the insecticides used in the control of the vectors, while behavioural resistance refers to changes that cause mosquitoes to avoid exposure [9, 16, 17]. Examples of behavioural adaptations include changing from late to early feeding, shifting from indoor to outdoor feeding, avoiding resting on LLINs or the walls sprayed with insecticides, and developing a preference to feed on non-human blood [13, 18–21].

The World Health Organization (WHO) recommends increased surveillance for changes in vector biology, physiology and behaviour following implementation of IRS and LLINs [22]. In response to these recommendations, scientists working in Uganda, where pyrethroid resistance is prevalent and likely to undermine the impact of LLINs, have extensively studied the physiological adaptations of mosquitoes to insecticides [7, 16, 23–25]. Findings from these studies have been used to inform the vector control policy in the country, including the rotation of insecticides to avoid prolonged selection for resistance, and the deployment of LLINs that have piperonyl-butoxide (PBO) in addition to pyrethroid insecticides to enhance their activity [26]. However, little has been done regarding surveillance for behavioural adaptations and changes in malaria vector species composition following the roll-out of vector-control interventions in the country. Effects of LLINs and IRS on *Anopheles* transmission intensity, biting behaviour and species composition in Tororo, a historically high malaria transmission area in Uganda were investigated. Results from this study will further inform roll-out of vector-control interventions in Uganda as the country moves towards achieving malaria control and eventual elimination as stipulated in the global technical strategy [27].

## Methods

### Study setting and population-level, malaria-control interventions

The study was carried out in Nagongera sub-county, Tororo district, eastern Uganda. Tororo is historically a high malaria transmission area with the *Plasmodium falciparum* annual entomological inoculation rate (*Pf*aEIR) estimated at 562 bites per person in 2001 and 125 in 2011–2012 [28, 29]. The area is characterized by savannah grassland interspersed with bare rocky outcrops and low-lying wetlands. There are typically two rainy seasons in this area with two peaks (May–June and November–December) with annual rainfall of 1000–1500 mm.

Malaria-control interventions in Tororo have included use of ACT for treatment of malaria, LLINs and IRS. Artemether-lumefantrine was adopted as first-line treatment for malaria in 2006 and is provided free of charge at the public health facilities. Tororo has had two rounds of universal free LLIN distribution, in November 2013 and June 2017, with the aim of delivering at least one LLIN for every 2 residents to over 90% of households [30]. Later studies show that 80% of the population used an LLIN among households that achieved universal coverage [31]. In December 2014, IRS with bendiocarb was introduced in Tororo for the first time. Although coverage of 90% was achieved in the district, some sub-counties such as Mulanda had as low as 78% coverage [32]. To date, the district has had 3 rounds of IRS with bendiocarb (December 2014–January 2015, June–July 2015, November–December 2015) and 4 rounds with Actellic (pirimiphos-methyl) (June–July 2016, July–August 2017, June–July 2018, March–April 2019).

### Study procedures

Data collection using human landing catches was carried out in two phases. The first phase was conducted from October 2011 to September 2012 before the introduction of the vector-control interventions, and the second phase was carried out between November 2017 and October 2018, 4 years after the roll-out of the combined vector control interventions. The procedures for data collection have been previously described [28]. Briefly, 8 households were randomly selected from a previously generated enumeration list. The selected households were visited prior to onset of the study procedures and consent for the household to participate in the study was sought from the head of the household. Four trained field workers and one field entomology supervisor visited each selected household on a monthly basis during the first phase of the study and every 4 weeks during the second phase of the study. Two of the field workers were stationed outdoors 10 m from the house between 18:00 and 22:00 h and two were stationed indoors between 18:00 and 06:00 h. The times for the indoor and outdoor catches were selected to replicate the normal human behaviour in the study area, with many residents outdoors in the early evening and most retiring to bed by 22.00. For each cycle of collection, 2 households were visited each night on 4 consecutive nights to ensure that all 8 households were sampled in the same week.

During mosquito collection, field workers who acted as human baits wore shorts to expose their legs, and sat on 40-cm high stools. Female mosquitoes that landed on the exposed legs of the field workers were collected using aspirators and flashlights. Specimens collected were placed in paper cups, covered with netting to prevent their escape, and delivered to the laboratory at Nagongera Health Centre the following morning. Individual mosquitoes were put in labelled micro-tubes and stored in zip-lock bags with a desiccant.

### Laboratory evaluations

Mosquitoes were sorted in the laboratory under a dissecting microscope to separate female *Anopheles* from male *Anopheles*, non-*Anopheles*, and other insects. Dichotomous keys were used to morphologically identify the different *Anopheles* species as previously described [33]. PCR was used to determine the species composition of *Anopheles gambiae* sensu lato (s.l.) complex using the protocol by Scott et al. [34]. Before vector-control interventions, a maximum of 10 mosquitoes per month were randomly selected for species determination. Since mosquito numbers markedly declined after initiation of the vector control interventions, species compositions of all *An. gambiae* s.l. were determined using PCR.

Infectivity of *Anopheles* with *P. falciparum* sporozoites was determined by enzyme-linked immunosorbent assay (ELISA). The head and thorax of each stored mosquito was separated from the rest of the body parts and ground in blocking buffer containing IGEPAL CA-630. An aliquot of 50 µl was transferred to plates coated with monoclonal antibodies and positive and negative controls added [35]. After a series of incubation and washing, the plate was read at 405–414 nm using an ELISA plate reader to differentiate between infected and non-infected mosquitoes.

### Calculation of annual entomological inoculation rate

The a*Pf*EIR, defined as the number of infective bites per person per year, was calculated as previously described [28]. Briefly, the *Pf*EIR was calculated using the following formula:

$$ {\text{a}}Pf{\text{EIR}} = {\text{HBR}} \times {\text{sporozoite}}\;{\text{rate}} \times 365\;{\text{days/year,}} $$

where HBR (the human biting rate) is the number of female *Anopheles* mosquitoes collected per house per night, and the sporozoite rate is the proportion of mosquitoes that tested positive for sporozoites.

### Data management and analysis

Data were double-entered into a Microsoft Access database and analysed using Stata (version 14.2, Stata Corp, College Station, TX, USA). The primary exposure was the combined vector control interventions (pre-intervention versus post-intervention). Outcomes of interest included the HBR, sporozoite rate, a*Pf*EIR, species compositions, and the proportion of mosquitoes collected outdoors. The HBR was further stratified based on whether mosquitoes were collected indoors or outdoors. Simple proportions were compared using a log-binomial regression model with generalized estimating equations to adjust for repeated measures from the same house. Incidence measures were compared using a negative binomial regression model with generalized estimating equations to adjust for repeated measures from the same house and measures of association expressed as the incidence rate ratio (incidence after the interventions/incidence before the interventions).

## Results

### Malaria transmission intensity

**Fig. 1:**
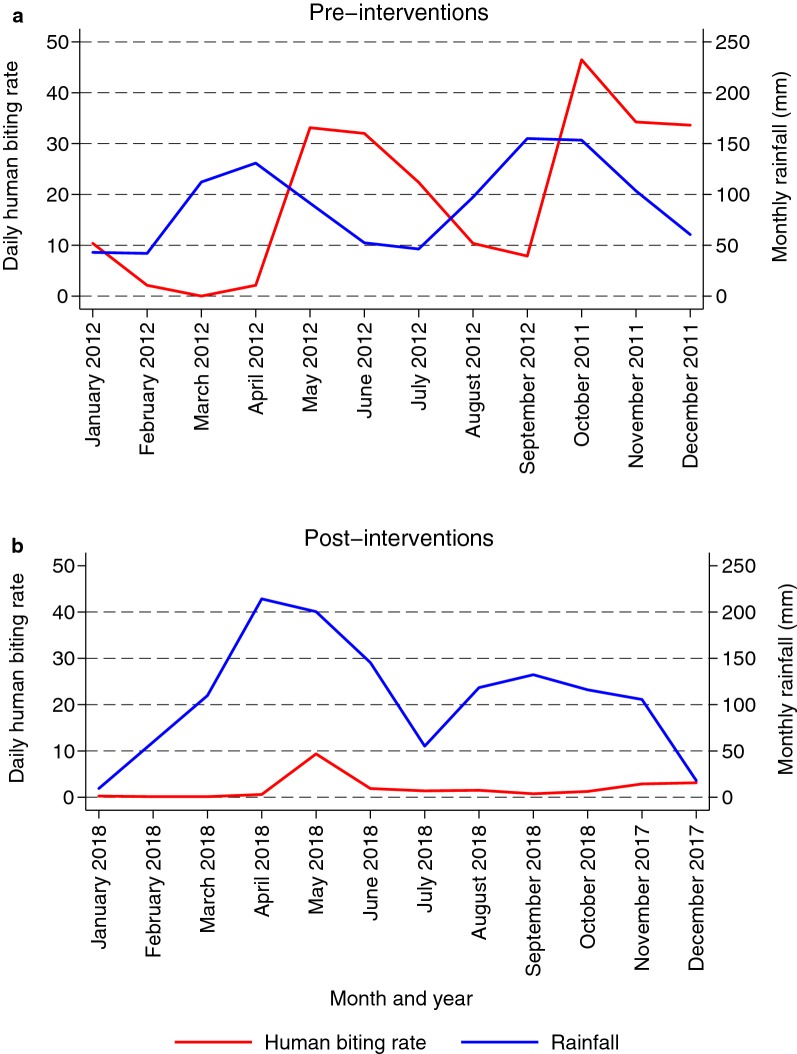
Monthly pattern of daily human biting rate by female *Anopheles* mosquitoes (red) and rainfall (blue) before and after intensive vector control interventions in Nagongera sub-county, Tororo district

Combination of IRS with LLINs was associated with an 88% reduction in the total HBR (from 19.6 to 2.3 bites per person per night, p < 0.001 Table 1). Stratification by source of the mosquitoes shows a higher decline in HBR in mosquitoes biting from indoors (17.3 to 1.2 bites per person per night) compared to those biting outdoors (2.3 to 1.2 mosquitoes per person per night) (risk difference (RD) = 16.1 for indoors versus RD = 1.1 for outdoors, p < 0.001). Accounting for rainfall, the daily HBR collapsed by month show low HBR following the large-scale deployment of vector control (Fig. 1). Before vector control 33/1878 (1.8%) of *Anopheles* samples tested positive for *P. falciparum* sporozoites, whilst after vector control none of the 243 mosquitoes sampled was sporozoite positive. Consequently, the a*Pf*EIR dropped from 129 to 0 infective bites per person per year (p < 0.001) following vector control. The Bayesian confidence interval for the proportion of mosquitoes that were sporozoite positive was 0.2% (0–0.78%); a confidence interval on the post-control *aPf*EIR was approximately 1.7 (0–6.5 infective bites, per person, per year).

**Table 1:** Impact of vector interventions on malaria transmission intensity

Metric	Pre-intervention	Post-intervention	Unadjusted		Adjusted^a^	
			IRR (95% CI)	p-value	IRR (95% CI)	p-value
Total human biting rate^b^	19.6	2.3	0.12 (0.08–0.17)	< 0.001	0.07 (0.04–0.11)	< 0.001
Indoor human biting rate^b^	17.3	1.2	0.07 (0.05–0.10)	< 0.001	0.03 (0.02–0.05)	< 0.001
Outdoor human biting rate^b^	2.3	1.2	0.51 (0.35–0.75)	0.001	0.34 (0.22–0.53)	0.001
Sporozoite rate	33/1878 (1.8%)	0/243 (0%)	N/A	< 0.001	N/A	< 0.001
aEIR^c^	129	0	N/A	< 0.001	N/A	< 0.001

*IRR* incidence rate ratio, *CI* confidence interval

^a^Adjusted for monthly rainfall with a 1 month lag time

^b^Number of female *Anopheles* collected per house per night

^c^Annual entomological inoculation rate

### Outdoor mosquito biting and time of biting

**Fig. 2:**
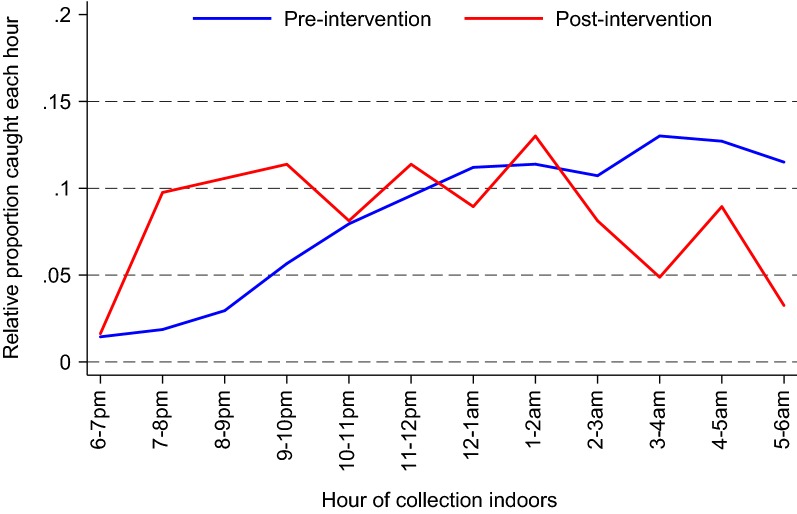
Proportions of hourly indoor mosquito catches before (blue) and after (red) intensive vector control interventions in Nagongera sub county, Tororo district

Increase in the proportion of mosquitoes collected outdoors from 11.6% before initiation of integrated vector control to 49.4% after initiation of integrated vector control was observed (p < 0.001). Overall, the number of *Anopheles* biting outdoors declined but they made up a larger proportion of the vector population after vector control than they did before initiation of vector control. Likewise, the proportion of mosquitoes collected indoors in the early evening (18.00 and 22.00) was higher after the interventions (198/1660 (11.9%) before interventions versus 41/123 (33.3%) after interventions, p < 0.001, Fig. 2).

### *Anopheles* mosquito species composition

Before vector-control interventions, 1878 *Anopheles* were collected and identified morphologically, of these 91.9% (1725) were *An. gambiae* s.l., 3.7% (70) were *Anopheles funestus* s.l. and 4.4% (83) were other *Anopheles*. After vector-control interventions, 243 *Anopheles* were collected, of these 79.8% (194) were *An. gambiae* s.l. and 20.2% (49) were other *Anopheles* species. No *An. funestus* was collected. To determine how interventions impacted on the relative prevalence of the species comprising the *gambiae* complex, PCR was used to determine the species of 296 *An. gambiae* s.l. (103 pre-intervention, 193 post-interventions). PCR amplification failed in 7 specimens (2.3%). Before vector-control interventions, 76.7% of *An. gambiae* s.l. were *An. gambiae* s.s. (79/103) and 23.3% were *Anopheles arabiensis* (24/103). Following vector-control interventions, 99.5% of *An. gambiae* s.l. species were *An. arabiensis* (193/194) and only a single *An. gambiae* s.s. was collected (1/194, 0.5%, Table 2). The biting intensity associated with *An. arabiensis* pre-intervention was 4.2 bites per night (i.e., 19.6 bites per night × 0.214), whilst after the intervention it was 1.8 bites per night (i.e., 2.3 bites per night × 0.794).

**Table 2:** Vector species composition before and after vector control

Intervention period	Species composition by morphology (n)	Number tested by PCR	Species composition by PCR (n)	Estimated proportion by morphology and PCR	
Pre-intervention	*An. gambiae* s.l. (1725)	103	*An. gambiae* s.s.(79)	*An. gambiae* s.s.	70.5%
			*An. arabiensis* (24)	*An. arabiensis*	21.4%
	*An. funestus* (70)	N/A		*An. funestus*	3.7%
	Other *Anopheles* species (83)			Other *Anopheles* species	4.4%
Post-intervention	*An. gambiae* s.l. (194)	194	*An. gambiae* s.s.(1)	*An. gambiae* s.s.	0.4%
			*An. arabiensis* (193)	*An. arabiensis*	79.4%
	*An. funestus* (0)	N/A		*An. funestus*	0%
	Other *Anopheles* species (49)			Other *Anopheles* species	20.2%

## Discussion

Large-scale deployment of LLINs and IRS in an area with historically high levels of malaria was associated with an eightfold reduction in vector densities as measured by human landing catches, an increased proportion of vector biting outdoors and a shift in vector dominance from *An. gambiae* s.s. to *An. arabiensis*. Vector control, including high coverage of LLINs, 3 rounds of IRS using bendiocarb, a carbamate insecticide, and 3 rounds of IRS using Actellic, an organophosphate insecticide, resulted in an 88% reduction in the HBR. There was a substantial reduction in both indoor and outdoor *Anopheles* HBR after over 5 years of mass distribution of LLINs and repeated rounds of IRS.

The combination of the interventions resulted in a 93% reduction in indoor biting by malaria vectors and a 49% reduction in outdoor biting. The observed reduction is explained by a Bayesian spatio-temporal model supporting the hypothesis that the sharp decline in vector numbers was associated with high LLIN coverage and IRS [36, 37] although it is unclear whether the effect was due to LLINs or IRS or both. In a related study, no changes in malaria were observed in the aftermath of mass distributions of LLINs, but then sharp declines were observed after IRS [6]. Indeed, both bendiocarb and Actellic are persistent and effective insecticides for use against mosquitoes [38, 39]. However, although LLINs alone have been shown to reduce biting rates of *Anopheles* and consequently transmission intensity in Uganda [40, 41], LLINs alone were not sufficient to suppress malaria in northern Uganda after withholding IRS [42]. It is possible the LLINs enhanced the effect of IRS, but it is impossible to say from these data.

Whilst the actual number of bites outdoors and indoors declined following vector control, the relative abundance of mosquitoes collected biting outdoors increased from 11.6% before vector-control interventions to 49.4% after vector-control interventions. There was a shift in the relative abundance of species, as well; 71.5% of mosquitoes were *Anopheles gambiae* sensu stricto (s.s.) before vector control, whilst after vector control 79.4% were *An. arabiensis*. In 2001–2002 and 2011–2012, the major malaria vector species reported in the area, in order of dominance, were *An. gambiae* s.s., *An. funestus* and *An. arabiensis* [16, 28, 29]. However, the massive killing associated with vector control resulted in only one *An. gambiae* s.s. being collected and no *An. funestus*. Other studies have also shown that *An. funestus*, which is highly endophilic, is susceptible to IRS [43–45]. This shift in vector composition has been reported previously with the massive deployment of LLINs or IRS [10–12, 46] and is associated with the preferential killing of the highly endophilic and anthropophilic *An. gambiae* s.s., which is replaced by the more exophilic and zoophilic *An. arabiensis* [47]. In East Africa it has been shown that massive deployment of LLINs has resulted in a change in the species composition of the vectors, with the once dominant indoor vectors *An. gambiae* s.s. being replaced by *An. arabiensis* [13, 15, 48]. In these cases, whilst the overall number of vectors declines, there are proportionately more *An. arabiensis* than *An. gambiae* s.s. Overall the number of *An. arabiensis* biting outdoors declines, but they make up a larger proportion of the vector population after vector control than they did before. *Anopheles arabiensis* is an efficient malaria vector and is capable of maintaining malaria transmission [21, 49, 50], and is likely to do so in the study area even at low levels.

Both LLINs and IRS target mosquitoes that enter houses, and will also kill a proportion of outdoor-biting mosquitoes that enter houses, as indicated by the decline in number of outdoor-biting mosquitoes during the study. Thus, at least a proportion of mosquitoes collected outdoors are likely to enter houses during their lifetime. Although mosquitoes were not collected outdoors throughout the night, there was an indication that indoor-biting vectors were biting earlier in the evening after the intervention than before the intervention. This slight shift to early evening has been associated with the massive deployment of LLINs in sub-Saharan Africa [51], with 21% of biting occurring before the time people are in bed, a percentage higher than previously recorded.

After vector control, none of the 243 potential malaria vectors tested positive for *P. falciparum* sporozoites, compared to 1.8% sporozoites rate before the interventions were deployed, suggesting that vector control markedly reduced infectivity of mosquitoes with *falciparum* sporozoites. These results are consistent with other studies that have shown a sharp reduction in sporozoites rates after vector control. For example, after 3 rounds of spraying with insecticides in Bioko Island, Equatorial Guinea, the sporozoite rate dropped from 8.3% before spraying to 0% after spraying with pyrethroid and carbamate insecticides [52]. Similarly in western Kenya, the sporozoite rate dropped from 3.4% before intervention to 0.8% after intervention with permethrin-treated bed nets [53]. These results suggest that high coverage and combinations of LLINs and IRS, where the mode of action of the insecticides on the walls and nets differ, are required to reduce the sporozoite rate.

Vector control was highly effective in the study area, but residual transmission begs the question, ‘What more needs to be done to eliminate malaria from the study area’? Addressing the issue of outdoor biting would help to further reduce malaria transmission, so mass drug administration or additional vector-based interventions that target outdoor biting could be considered [14, 54, 55], such as environmental management and larviciding, the use of insecticides on cattle, toxic sugar baits traps, spatial repellents, or transgenic mosquitoes [56, 57].

### Limitations of the study

The study was not designed to evaluate the effectiveness of the interventions in comparison with absence of such interventions since no similar entomological data were collected in other areas without such interventions.

## Conclusions

This study has shown that high coverage of LLINs and IRS combined over 5 years markedly reduced malaria transmission in an area with historically high burdens of malaria. The combination of tools aimed at endophilic vectors reduced the number of vectors biting indoors and outdoors, but increased the proportion of outdoor biting and shifted the species composition of *An. gambiae* s.l. from predominantly *An. gambiae* s.s. to *An. arabiensis*. These findings demonstrate the effectiveness of vector control based on the use of insecticides indoors in areas where vectors are highly endophilic. Despite the considerable reduction in malaria in this area, elimination was not achieved and supplementary measures will be required to achieve that goal.

## Data Availability

Data are available upon reasonable request by an email to the corresponding author.

## Abbreviations

ACT: artemisinin-based combination therapy
aEIR: annual entomological inoculation rates
CSP: circumsporozoite protein
ELISA: enzyme-linked immunosorbent assay
GPS: global positioning systems
HBR: human biting rate
IPTp: intermittent preventive therapy in pregnancy
IRS: indoor residual spraying
LLIN: long-lasting insecticidal nets
PBO: piperonyl butoxide
PCR: polymerase chain reaction
*Pf*aEIR: *Plasmodium falciparum* annual entomological inoculation rate
RDTs: rapid diagnostic tests
TPR: test positivity rate
WHO: World Health Organization
